# The Role of Caspase-4 and NLRP1 in MCF7 Cell Pyroptosis Induced by hUCMSC-Secreted Factors

**DOI:** 10.1155/2020/8867115

**Published:** 2020-07-09

**Authors:** Yang Jiao, Linlin Wang, Lin Lu, Jianjun Liu, Xin Li, Hongbo Zhao, Zongliu Hou, Bingrong Zheng

**Affiliations:** ^1^State Key Laboratory for Conservation and Utilization of Bio-Resources in Yunnan & School of Medicine, Yunnan University, Kunming, 650091 Yunnan, China; ^2^Yunnan Key Laboratory of Stem Cell and Regenerative Medicine, Biomedical Engineering Research Center, Kunming Medical University, Kunming, 650500 Yunnan, China; ^3^Yan'an Hospital of Kunming City, Kunming, 650051 Yunnan, China

## Abstract

Mesenchymal stem cells (MSCs) are being widely investigated for the development of novel therapeutic approaches for different cancers, including breast cancer, the leading form of cancer in women. Our previous study showed that the factors secreted by human umbilical cord MSCs (hUCMSCs) induced pyroptosis in the breast cancer cell line MCF7 and our RNA sequencing studies revealed an increase in the expression of the pyroptosis-related gene caspase-4 (*CASP4*) and nucleotide-binding, leucine-rich repeat pyrin domain-containing protein 1 (*NLRP1*) in pyroptotic MCF7 cells. Cellular pyroptosis can occur via the canonical pathway (involving caspase-1 and NLRP1) or the noncanonical pathway (involving caspase-4). In this study, we first confirmed that the inflammasome complex formed by NLRP1 and ASC is involved in MCF7 cell pyroptosis induced by hUCMSC-CM. Further, we investigated the role of *CASP4* and *NLRP1* in MCF7 cell pyroptosis induced by hUCMSC-secreted factors using shRNA-mediated transfection of *CASP4* or *NLRP1* in MCF7 cells. Cytotoxicity analyses revealed that neither *CASP4* knockdown nor NLRP1 knockdown could inhibit the hUCMSC-CM-induced pyroptosis in MCF7 cells. Gene and protein expression analysis showed that hUCMSC-CM induced pyroptosis mainly via the canonical pathway in *CASP4* knockdown MCF7 cells but mainly via the noncanonical pathway in *NLRP1* knockdown MCF7 cells. Our study provides a foundation for further studies aimed at elucidating the precise mechanism underlying hUCMSC-induced pyroptosis in breast cancer cells and aid the identification of potential therapeutic targets for breast cancer.

## 1. Introduction

Pyroptosis, a type of programmed cell death accompanied with the release of several proinflammatory factors, plays an important role in immune response against infection. The morphological changes associated with pyroptosis involve pore formation in the plasma membrane, water influx, cell swelling, and the subsequent rupture of the plasma membrane and release of intracellular proinflammatory molecules [1]. In humans, pyroptosis is mediated by inflammatory caspases (caspase-1, caspase-4, and caspase-5), which may be activated by inflammasomes. The inflammasome pathways include the caspase-1-dependent canonical pathway and caspase-1-independent noncanonical pathway [2]. Caspase-1 activation induces gasdermin D cleavage, thereby leading to pore formation in the cell membrane and the maturation and release of IL-1*β* and IL-18 cytokines, which induce pyroptosis [3]. Nucleotide-binding, leucine-rich repeat pyrin domain-containing protein 1 (NLRP1), a member of NOD-like receptor (NLR) family, is an important natural immune molecule [4]. In humans, it activates pro-caspase-1 directly by interacting with it or indirectly by recruiting the adaptor protein ASC and pro-caspase-1 to form an inflammasome [5]. Therefore, NLRP1 plays an important role in cell pyroptosis mediated by the canonical pathway. The noncanonical pathway in humans involves the activation of caspase-4/caspase-5 [2].Caspase-4/caspase-5 cleaves gasdermin D, thereby triggering pyroptosis [6]. In human macrophages, caspase-4 activation by *Legionella pneumophila* induced cell death and IL-1*α* secretion [7]. Intracellular lipopolysaccharide (LPS) directly interacts with caspase-4 and induces cell pyroptosis [8].

Breast cancer is the leading type of cancer among women [9], and rising breast cancer incidence has been reported in China [10]. However, an effective treatment for breast cancer is not yet available. Mesenchymal stem cell- (MSC-) based approaches are being studied extensively for the development of new cancer therapeutic strategies. Human umbilical cord mesenchymal stem cells (hUCMSCs) are widely used in research focused on cancer treatment owing to their easy availability and no ethical issues [11–13]. We previously demonstrated that the factors secreted by hUCMSCs induced pyroptosis in the breast cancer cell line MCF7.Furthermore, RNA sequencing studies revealed a significant increase in the expression of pyroptosis-related genes *CASP4* and *NLRP1* in pyroptotic MCF7cells [14]. Thus, caspase-4 and NLRP1 may play a role in this process. Although some of the mechanisms underlying the function of *NLRP1* and *CASP4* in pyroptosis are known, the effects of these two genes in MCF7 cell pyroptosis induced by hUCMSC-secreted factors remain unclear. Therefore, in the present study, we elucidated the role of caspase-4 and NLRP1 on MCF7 cell pyroptosis induced by hUCMSC-secreted factors. Our study provides the possible mechanism underlying hUCMSC-induced pyroptosis in breast cancer cells and may provide potential therapeutic targets for breast cancer.

## 2. Materials and Methods

### 2.1. Cell Culture

The breast cancer cell line MCF7 (Kunming Cell Bank of the Chinese Academy of Sciences, Kunming, China) was maintained in Dulbecco's modified Eagle's medium (DMEM; Gibco by Thermo Fisher Scientific™, Suzhou, China) containing l-glutamine, 4.5 g/L glucose, and 110 mg/L sodium pyruvate and supplemented with 10% MSC-qualified fetal bovine serum (FBS; Biological Industries, Australia), 100 mg/L streptomycin, and 100 mg/L penicillin (Gibco by Thermo Fisher Scientific™, NY, USA) at 37°C with 5% CO_2_.

The hUCMSCs were isolated from the human umbilical cord Wharton jelly and identified as described previously [14]. The study was approved by the Medical Ethics Committee of Yunnan University Medical School, and informed consent was obtained from all the donors. hUCMSCs were cultured in minimum essential medium alpha modification (*α*MEM; HyClone by GE Healthcare, Beijing, China) supplemented with 10% MSC-qualified FBS, 100 mg/L streptomycin, 100 mg/L penicillin, and 10 ng/mL basic fibroblast growth factor (bFGF; Merck Millipore, Darmstadt, Germany) at 37°C with 5% CO_2_. The hUCMSCs were used before the eighth passage.

### 2.2. shRNA Vectors

Four different sets pGPH1/GFP/Neo vectors (Shanghai GeneChem Co., Ltd., Shanghai, China) expressing *CASP4* or *NLRP1*shRNA were used for *CASP4* or *NLRP1* knockdown. The sequences used for shRNA-mediated knockdown are listed in Table 1. The shRNA vectors were identified by sequencing, and the successful insertion of the target sequence in pGPH1/GFP/Neo vectors and the accuracy of the nucleotide sequences was confirmed (Additional file 1).

### 2.3. Exposure of MCF7 Cells to hUCMSC-Conditioned Medium (hUCMSC-CM)

hUCMSCs were cultured in plastic flasks (25 cm^2^; Corning, NY, USA). At ~90% confluency, the cultured medium was collected and filter sterilized using a 0.22 *μ*m Millex-GP Filter Unit (Millipore, Carrigtwohill, Ireland). Conditioned medium (CM) was prepared using 80% hUCMSC-cultured medium and 20% fresh medium, as described previously [14]. MCF7 cells were seeded in 6-well plates (Corning) at a density of 1 × 10^5^ cells/mL in normal medium and cultured overnight. Then, the cells were transfected with 2.5 *μ*g shRNA expression vectors per well using Lipofectamine 3000 reagent for 72 h. The medium was then replaced with hUCMSC-CM.

### 2.4. ASC Speck Staining

MCF7 cells were seeded on cover slips in a 24-well plate (Corning, NY, USA) containing normal medium and cultured overnight. Then, the medium was replaced with hUCMSC-CM. After 24 h, the cells were fixed with 4% paraformaldehyde, permeabilized with 0.1% Triton X-100, and blocked with PBS buffer containing 5% BSA. Cells were stained with anti-ASC antibody (1 : 100; Proteintech, Wuhan, China) and AlexaFluor488-conjugated secondary antibody (1 : 200; Thermo Fisher Scientific, Shanghai, China), and with anti-NLRP1 antibody (1 : 100; Santa Cruz Biotechnology) and AlexaFluor594-conjugated secondary antibody (1 : 200; Thermo Fisher Scientific, Shanghai, China). DAPI was used to stain nuclei. Cell images were captured using inverted phase contrast optics (Leica DFC420C).

### 2.5. Annexin V/Propidium Iodide (PI) Analysis

MCF7 cells were collected at 72 h after transfection and cultured in hUCMSC-CM for 24 h. The percentage of dead cells was determined using the Annexin V-FITC/PI apoptosis detection kit (CWBio, Beijing, China). Briefly, cells were collected after trypsin digestion without EDTA and washed three times with cold PBS. The cells were resuspended in a binding buffer at a density of 1 × 10^6^ cells/mL, incubated with 10 *μ*L PI and 5 *μ*L Annexin V-fluorescein isothiocyanate (FITC) for 10 min at 37°C, and analyzed using the CyFlow Space flow cytometer (Sysmex Partec) and FloMax 2.82 software (Sysmex Partec).

### 2.6. LDH Cytotoxicity Assay

The degree of cell damage was determined using LDH-Glo™ Cytotoxicity Assay (Promega, Beijing, China). MCF7 cells were collected at 48 h after transfection, seeded in 96-well plates (Corning) at a density of 5 × 10^3^ cells/mL, and cultured overnight. The medium was replaced with hUCMSC-CM, and the cells were cultured for 24 h. Then, 5 *μ*L cultured medium was added into 95 *μ*L LDH Storage Buffer, and the resulting solution was diluted five times using the LDH Storage Buffer. Diluted standard solutions were prepared per the manufacturer's instructions. Then, 50 *μ*L sample/standard was incubated with 50 *μ*L LDH Detection Reagent in each well of an opaque 96-well plate at 20–25°C for 1 h, and the luminescence was recorded using the Modulus Microplate Multimode Reader (Turner Biosystems, California, USA).

### 2.7. Reverse Transcription Quantitative Real-Time Polymerase Chain Reaction (RT-qPCR)

MCF7 cells were collected at 72 h after transfection and cultured in hUCMSC-CM for 24 h. Total RNA was extracted using the TRIzol™ reagent (Invitrogen, Carlsbad, CA, USA). The quantity and quality of RNA were assessed using the Nano-300 spectrophotometer (Hangzhou Allsheng Instruments Co., Ltd., Hangzhou, China). First-stand cDNA was synthesized using the PrimeScript™ RT Reagent Kit with gDNA Eraser (Takara Bio., Beijing, China). The primer sequences were designed using the PrimerQuest Tool (http://http://www.idtdna.com); the sequences are listed in Table 2. q-PCR was performed using FastStart Universal SYBR Green Master (Roche, Mannheim, Germany) and Bio-Rad CFX96™ Real-Time PCR Detection System (Bio-Rad, Shanghai, China). Relative quantification was performed using the comparative Ct (2^-*ΔΔ*Ct^) method [15].

### 2.8. Western Blotting

MCF7 cells transfected for 72 h and cultured in hUCMSC-CM for 24 h were lysed in 50 *μ*L RIPA lysis buffer (strong) containing 0.5 *μ*L phenylmethylsufonyl fluoride (CWBio, Jiangsu, China) and incubated on ice for 2 h. The lysed cells were incubated with 50 *μ*L of 2x SDS-PAGE protein loading buffer (Bio-Rad) in boiling water for 10 min. After centrifugation, the protein samples were subjected to 10% SDS-PAGE and transferred onto polyvinylidene difluoride membranes (Merck Millipore). The membranes were blocked in TBST (3.0 g Tris-HCl, 8.0 g NaCl, 0.1% Tween-20, and pH 7.4) containing 5% nonfat dried milk for 1 h and incubated overnight at 4°C with diluted primary antibodies against GAPDH (1 : 2000; CWBio, Jiangsu, China), caspase-1 (1 : 500; Santa Cruz Biotechnology, Delaware, USA), NLRP1 (1 : 100; Santa Cruz Biotechnology), caspase-4 (1 : 500; Proteintech, Wuhan, China), and ASC (1 : 1000; Proteintech, Wuhan, China). The membranes were incubated with horseradish peroxidase-conjugated goat anti-mouse IgG or goat anti-rabbit IgG (1 : 2000; CWBio, Jiangsu, China) at 20–25°C for 1 h and then with ECL substrate solution (1 : 1 (*v*/*v*); CWBio, Jiangsu, China). The bands were quantified using Photoshop 7.0, and the gray value ratio of bands was determined.

### 2.9. ELISA-Based Quantification of Secreted IL-*α*, IL-1*β*, and IL-18

Media from MCF7 cells transfected with shRNA vectors for 72 h (control group) and media from MCF7 cells transfected with shRNA vectors for 72 h and cultured in hUCMSC-CM for 24 h (treatment group) were collected. All the samples were stored at -80°C before detection. The amounts of IL-*α*, IL-1*β*, and IL-18 secreted by MCF7 cells in the control and treatment groups were determined using the Human IL-*α* ELISA Kit (ExCell Biotech, Jiangsu, China), Human IL-1*β*/IL-1F2 Valukine™ ELISA Kit (NOVUS Biologicals, Taiwan, China), and Human IL-18 Kit (OriGene, Rockville, MD, USA) separately, according to the manufacturer's instructions.

### 2.10. Statistical Analyses

Statistical analyses were performed using Microsoft Excel 2007 and GraphPad Prism 5 software; graphs were prepared using the GraphPad Prism 5 software. Differences with *P* values *<* 0.05 were considered statistically significant.

## 3. Results

### 3.1. Effect of hUCMSC-CM Treatment on mRNA and Protein Levels of Caspase-1, Caspase-4, and NLRP1 in MCF7 Cells

**Figure 1:**
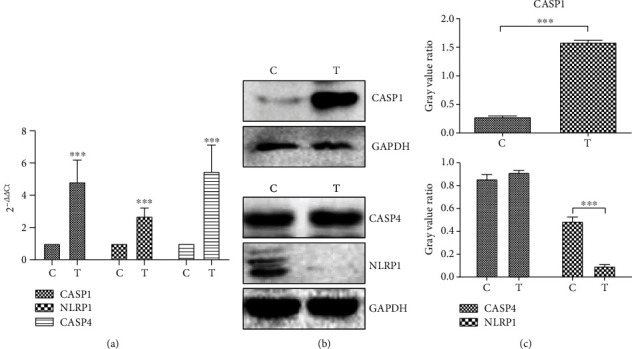
Detection of pyroptosis-related genes. (a) The results of q-PCR. Data are presented as Ct (2^-*△△*Ct^) relative to control. Data are presented as mean ± S.D., *n* = 3. (b) The results of western blotting. (c) The gray value ratio of western blotting results. C: control group, MCF7 cells cultured in the hUCMSC medium; T: treatment group, MCF7 cells cultured in hUCMSC-CM medium. ^∗^*P* < 0.05, ^∗∗^*P* < 0.01, and ^∗∗∗^*P* < 0.001.

We previously demonstrated that the factors secreted by hUCMSCs induced pyroptosis in MCF7 cells and that such pyroptotic cells showed significantly increased CASP4 and NLRP1 expression [14]. To further confirm this result, we analyzed the expression of *CASP1*, *CASP4*, and *NLRP1*in MCF7 cells cultured in hUCMSC-CM for 48 h. RT-qPCR analysis showed that *CASP1*, *CASP4*, and *NLRP1* mRNA levels in MCF7 cells cultured in hUCMSC-CM were significantly higher (fold increase of 5.16 ± 1.92, 5.48 ± 2.62, and 2.89 ± 0.79, respectively) than those in the control cells (*P* < 0.001; Figure 1(a)). These results were consistent with our previous RNA sequencing results.

In order to know whether the changes of mRNA level affect the protein expression, we did the western blotting analysis. Compared to the control cells, the cells cultured in hUCMSC-CM showed a significant increase in CASP1 protein levels (*P* < 0.001), no significant change in CASP4 protein levels (*P* > 0.05), and significant decrease in NLRP1 protein levels (*P* < 0.001; Figures 1(b) and 1(c)). These results indicate that the changes in protein levels of these three molecules are not consistent with the changes in their respective mRNA levels. Caspase-1 usually exists in cells in the inactive form as pro-caspase-1. In response to cellular stress or microbial infection, pro-caspase-1 is cleaved to p10 and p20 subunits, and the activated caspase-1 is a tetramer composed of two p20 and two p10 subunits (p20/p10) [16]. Caspase-1 recruitment to inflammatory signaling hubs was reported to enable its activation likely by increasing the local concentration of pro-caspase-1 to facilitate the dimerization of the monomers [17]. Full-length caspase-1 monomers can undergo dimerization, activation, and self-cleavage only when they are recruited to the inflammasomes; this enables a high local concentration of monomers [18]. In our study, pyroptotic MCF7 cells showed a significant increase in CASP1 protein levels, which is consistent with these theories. Similar to caspase-1, caspase-4 exists in cells in an inactive form and its active form is a tetramer composed of two p20 and two p10 subunits (p20/p10). In the present study, pyroptotic MCF7 cells showed increased *CASP4* mRNA but not protein levels, possibly because of homeostasis, indicating that caspase-4 activation requires pro-caspase-4 consumption, and the increase in *CASP4* mRNA replenished the procaspase-4 amount consumed. NLRP1 interacts with the adaptor protein ASC to form an inflammasome complex, which recruits pro-caspase-1 [5]. Therefore, reduced NLRP1 protein levels observed in this study may be because the NLRP1 protein molecules were consumed for formation of the NLRP1 inflammasome, and these molecules were not detected by the antibody used in western blotting.

### 3.2. Effect of Inflammasome Complex Formed by NLRP1 and ASC on hUCMSC-CM-Induced Pyroptosis in MCF7 Cells

**Figure 2:**
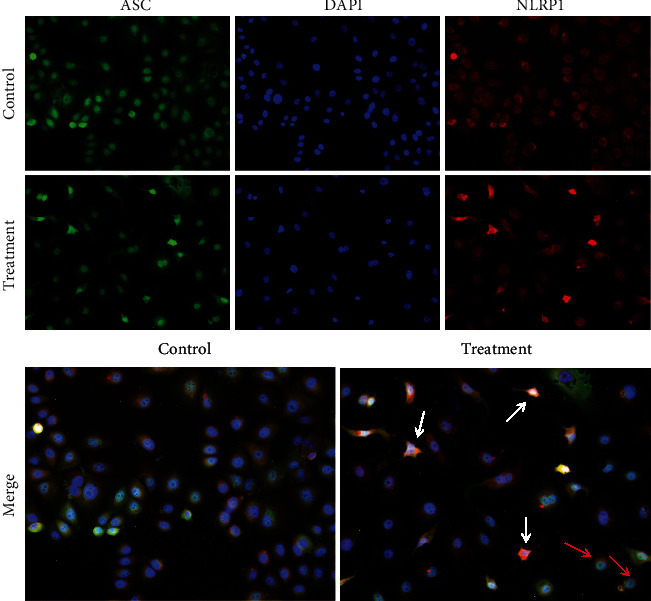
The immunofluorescence images of ASC specks. Representative micrographs from at least three independent experiments are shown. All images were taken using 20x magnification.

Next, we performed immunofluorescence analysis to investigate whether NLRP1 interacts with the adaptor protein ASC to form an inflammasome complex in pyroptotic MCF7 cells induced by hUCMSC-CM. Colocalization of NLRP1 protein and ASC protein to form a complex was observed in certain parts of some MCF7 cells cultured in hUCMSC-CM (red arrow, Figure 2), and strong colocalization and increased fluorescence intensities of both NLRP1 and ASC proteins were observed in pyroptotic cells (white arrow, Figure 2). These results indicate that NLRP1 could interact with ASC to form an inflammasome complex and that this complex is involved in hUCMSC-CM-induced pyroptosis in MCF7 cells.

### 3.3. Effect of *CASP4* or NLRP1 Knockdown on hUCMSC-CM-Induced Pyroptosis in MCF7 Cells

On the basis of RT-qPCR analysis performed to assess the inhibition rate of shRNA vectors, the vectors shRNA-*CASP4*-100, shRNA-*CASP4*-1104, shRNA-*NLRP1*-1634, and shRNA-*NLRP1*-2523 were selected for the transfection experiments (Additional file 2).

**Figure 3:**
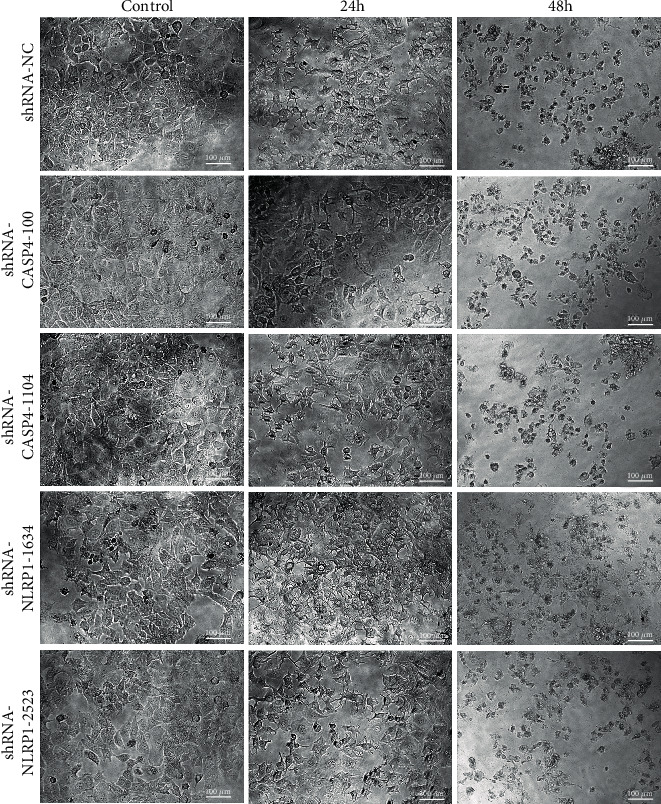
Morphological changes in transfected MCF7 cells exposed to hUCMSC-CM. Images were obtained on 24 h and 48 h. Representative micrographs from at least three independent experiments are shown. All images were taken using 20x magnification.

We first observed the morphological changes in the transfected MCF7 cells cultured in hUCMSC-CM. The plasma membrane of these cells remained intact but showed pore-induced invaginations. Distinctly ruptured plasma membrane and cell death were observed (Figure 3). These morphological changes indicate the occurrence of pyroptosis in transfected MCF7 cells after treatment with hUCMSC-CM.

**Figure 4:**
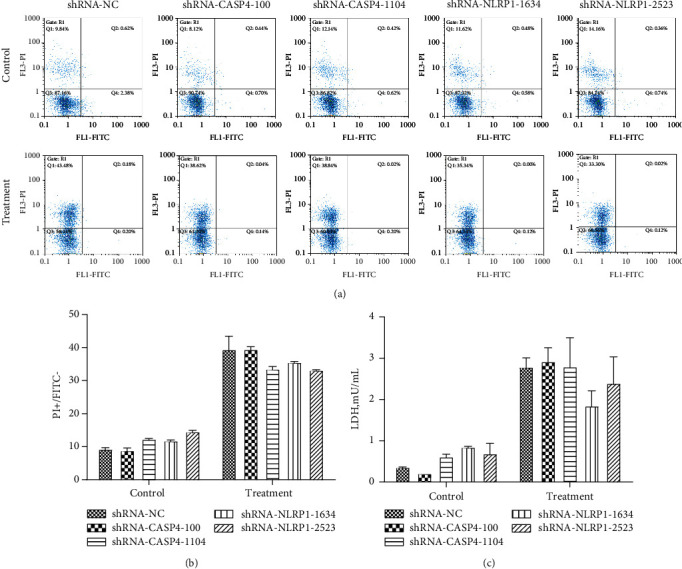
Detection of dead cells. (a) Flow cytometry results of Annexin V-FITC/PI staining. (b) Quantitative analysis for the Annexin V-FITC/PI results. (c) The concentration of LDH. Control: cells were collected after transfection with shRNA vector for 72 h. Treatment: cells were collected after transfected with shRNA vector for 72 h and treated with hUCMSC-CM for 24 h. Data were presented as mean ± SD of three independent experiments.

To understand whether cell death decreased after *CASP4* or *NLRP1* knockdown, we performed the Annexin V-FITC/PI analysis in *CASP4* knockdown and *NLRP1* knockdown MCF7 cells. The numbers of FITC^+^/PI^+^ cells and FITC^+^/PI^–^ cells remained unchanged after hUCMSC-CM treatment. However, the number of PI^+^/FITC^–^ cells among the transfected cells increased significantly after treatment with hUCMSC-CM for 24 h. The numbers increased from 9.27 ± 0.60 to 39.42 ± 5.74, 8.86 ± 1.05 to 39.46 ± 1.18, 12.30 ± 0.22 to 33.55 ± 1.02, 11.81 ± 0.27 to 35.57 ± 0.33, and 14.56 ± 0.60 to 33.20 ± 0.14 in cells transfected with shRNA-NC (negative control), shRNA-*CASP4*-100, shRNA-*CASP4*-1104, shRNA-*NLRP1*-1634, and shRNA-*NLRP1*-2523, respectively (Figures 4(a) and 4(b)). The number of apoptotic cells in the groups transfected with the target genes was not significantly different from that in the NC group (*P* > 0.05). These results suggest that hUCMSC-CM treatment for 24 h induced pyroptosis in all the transfected MCF7 cell groups, including the cells transfected with NC. Thus, MCF7 cells cannot be rescued from hUCMSC-CM-induced pyroptosis by inhibiting the expression of *CASP4* or *NLRP1*.

To further confirm these results, we assessed cytotoxicity by measuring the LDH levels in the medium of the transfected MCF7 cells treated with hUCMSC-CM. The LDH levels in all the cell groups, including the NC group, increased after treatment with hUCMSC-CM for 24 h; however, no significant difference was observed between the LDH levels of the target gene-transfected groups and the NC group (Figure 4(c)). These results are consistent with those of Annexin V-FITC/PI analysis and indicate that *CASP4* or *NLRP1* inhibition could not prevent hUCMSC-CM-induced pyroptosis in MCF7 cells.

### 3.4. Effect of hUCMSC-CM Treatment on Gene Expression in Transfected MCF7 Cells

**Figure 5:**
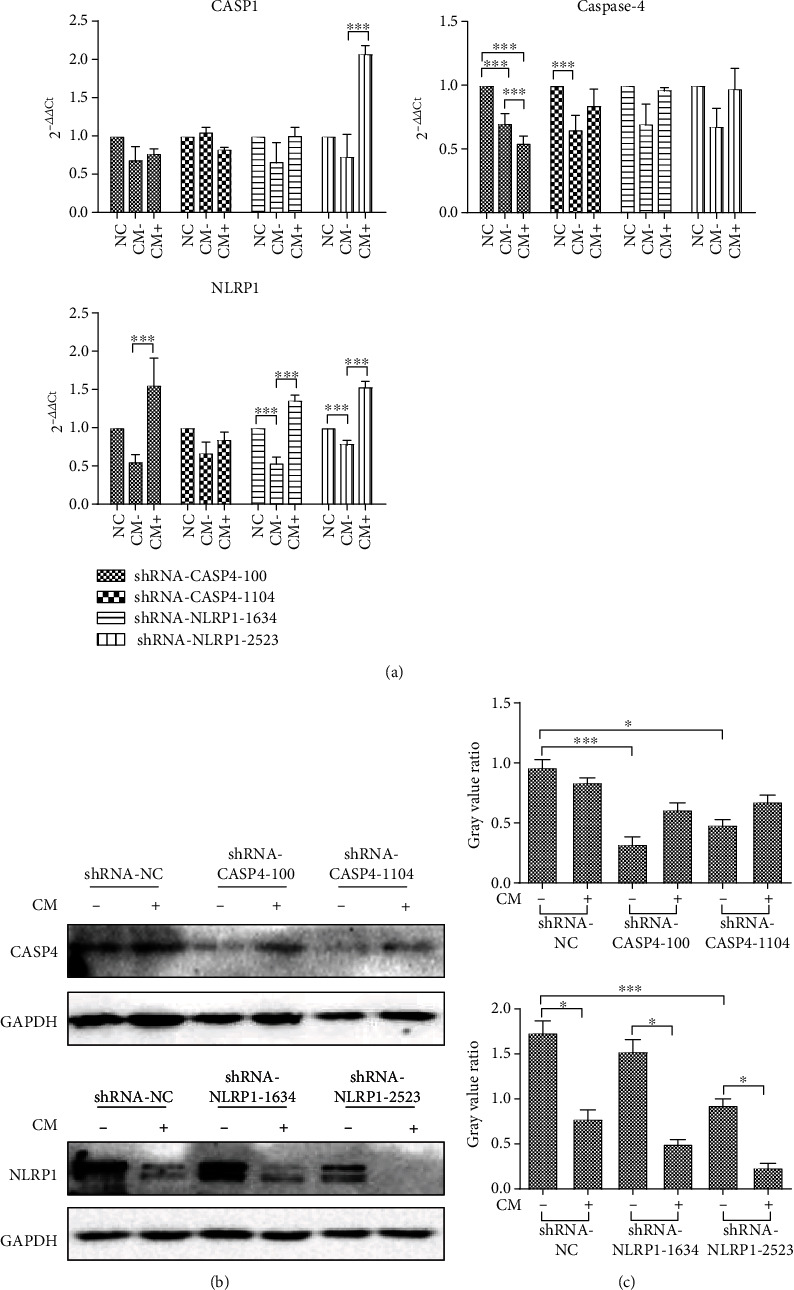
Detection of genes expression. (a) The q-PCR results of pyroptosis-related genes. Data are presented as Ct (2^-*△△*Ct^) relative to control. Data are presented as mean ± S.D., *n* = 3. ^∗^*P* < 0.05, ^∗∗^*P* < 0.01, and ^∗∗∗^*P* < 0.001. (b) The results of western blotting. (c) The gray value ratio of western blotting results. NC: negative control. CM-: cells were collected after transfection with shRNA vector for 72 h. CM+: cells were collected after transfection with shRNA vector for 72 h and treated with hUCMSC-CM for 24 h. ^∗^*P* < 0.05, ^∗∗^*P* < 0.01, and ^∗∗∗^*P* < 0.001.

Although inhibition of *CASP4* or *NLRP1* gene expression did not suppress the effect of hUCMSC-CM on inducing MCF7 cell death, we attempted to elucidate the mechanism underlying pyroptosis induction in MCF7 cells. Here, we analyzed the mRNA levels for caspase-1, caspase-4, and NLRP1 in *CASP4* knockdown, *NLRP1* knockdown, and NC-transfected cells treated with hUCMSC-CM. RT-qPCR analysis (Figure 5(a)) revealed a significant decrease in *CASP4* mRNA levels in the hUCMSC-CM-treated *CASP4* knockdown MCF7 cells, compared to the NC-transfected MCF cells (*P* < 0.001); however, no considerable change was observed in the *CASP1* mRNA levels of the *CASP4* knockdown MCF7 cells (*P* > 0.05). Furthermore, treatment with hUCMSC-CM significantly increased the *NLRP1* mRNA levels in MCF7 cells transfected with shRNA-*CASP4*-100 (*P* < 0.001). The *NLRP1* mRNA levels in *NLRP1* knockdown MCF7 cells showed a significant decrease initially (*P* < 0.001) but increased after treatment with hUCMSC-CM (*P* < 0.001). *CASP4* mRNA levels in *NLRP1* knockdown MCF7 cells did not change considerably (*P* > 0.05); however, treatment with hUCMSC-CM significantly increased the *CASP1* mRNA levels in MCF7 cells transfected with shRNA-*NLRP1*-2523 (*P* < 0.001).

To further know the changes of protein level in transfected MCF7 cells, we performed western blotting. Western blotting results (Figures 5(b) and 5(c)) revealed significantly decreased caspase-4 protein levels in *CASP4* knockdown MCF7 cells (*P* < 0.05); however, the decrease was more prominent in cells transfected with shRNA-*CASP4*-100 (*P* < 0.001). Compared to NC-transfected MCF7 cells, the MCF7 cells transfected with shRNA-*NLRP1*-2523 showed a significant decrease in NLRP1 protein levels (*P* < 0.05), and hUCMSC-CM treatment further decreased the NLRP1 protein levels in these cells. Therefore, shRNA-*CASP4*-100 and shRNA-*NLRP1*-2523 are more effective for gene inhibition, and we selected MCF7 cells transfected with shRNA-*CASP4*-100 and shRNA-*NLRP1*-2523 for further research.

### 3.5. Involvement of the Two Pyroptosis Pathways in hUCMSC-CM-Induced Pyroptosis in MCF7 Cells

To elucidate the effect of *CASP4* or *NLRP1* knockdown on MCF7 cell pyroptosis, we investigated the protein levels of pro-CASP1, cleaved CASP1, pro-CASP4, and cleaved CASP4 and the changes in ASC speck formation in MCF7 cells transfected with shRNA-*CASP4*-100 and shRNA-*NLRP1*-2523. In *CASP4* knockdown MCF7 cells, hUCMSC-CM treatment for 24 h significantly decreased the levels of cleaved CASP4 but significantly increased the levels ofcleaved CASP1, ASC, and NLRP1. These findings indicate that the noncanonical pathway was inhibited in *CASP4* knockdown MCF7 cells and that hUCMSC-CM-induced pyroptosis mainly occurs via the caspase-1-mediated canonical pathway. Conversely, 24 h treatment with hUCMSC-CM in *NLRP1* knockdown MCF7 cells did not affect the levels of cleaved CASP4 and pro-CASP1 but significantly decreased the levels of cleaved CASP1. This indicates that *NLRP1* inhibition did not affect the noncanonical pathway but could reduce cleaved CASP1 protein levels. Furthermore, the ASC speck in *NLRP1* knockdown cells was significantly lower than that in *CASP4* knockdown cells, indicating the inhibition of inflammasome formation and the subsequent partial inhibition of the canonical pathway in *NLRP1* knockdown cells. Therefore, the canonical pathway was inhibited in *NLRP1* knockdown MCF7 cells, and hUCMSC-CM-induced pyroptosis in these cells mainly occurs via the caspase-4-mediated noncanonical pathway.

**Figure 6:**
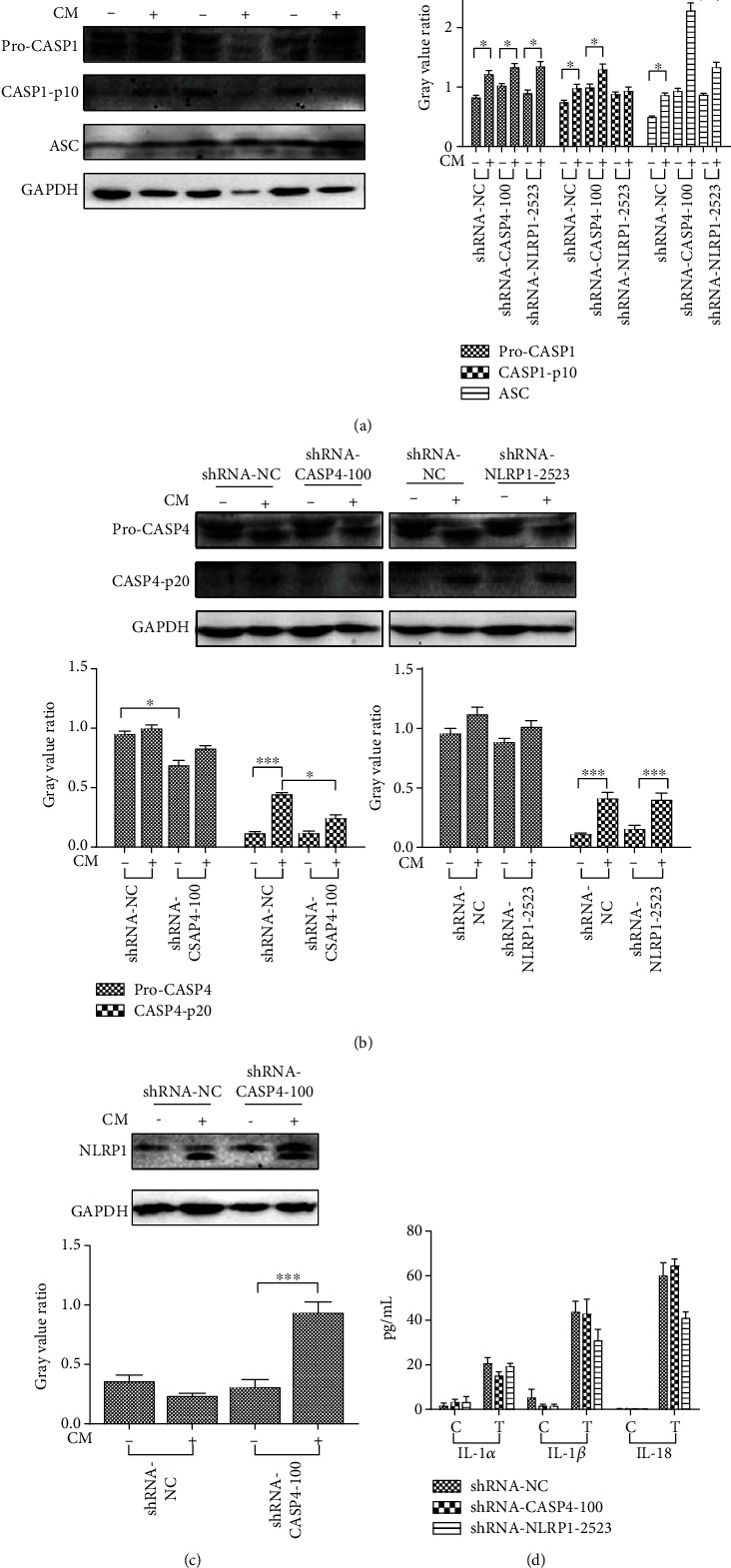
Study on the effect of *CASP4* and NLRP1 genes on MCF7 pyroptosis induced by hUCMSC-CM. (a) The western blotting results of pro-CASP1, cleaved-CASP1, and ASC proteins. (b) The western blotting results of pro-CASP4 and cleaved-CASP4. (c) The western blotting results of NLRP1. (d) The concentration of secreted cytokine in cell supernatant. NC: negative control. CM-: cells were collected after transfection with shRNA vector for 72 h. CM+: cells were collected after transfection with shRNA vector for 72 h and treated with hUCMSC-CM for 24 h. C: cells were collected after transfection with shRNA vector for 72 h. T: cells were collected after transfection with shRNA vector for 72 h and treated with hUCMSC-CM for 24 h. Data were presented as mean ± SD of three independent experiments.^∗^*P* < 0.05, ^∗∗^*P* < 0.01, and ^∗∗∗^*P* < 0.001.

IL-1*α* secretion is caspase-1 independent [19] but is positively correlated with the activity of caspase-4 [7, 20], a key factor of the noncanonical pathway. The secretion of IL-1*β* and IL-18 is positively correlated with the activity of caspase-1, a key factor of the canonical pathway [21, 22]. Therefore, we assessed the levels of IL-1*α*, IL-1*β*, and IL-18 in the culture medium of transfected MCF7 cells treated with hUCMSC-CM for 24 h to determine the involvement of the noncanonical and canonical pathways in hUCMSC-CM-induced pyroptosis in MCF7 cells in the absence of caspase-4 or NLRP1, respectively. The control groups showed low secretion of all the three cytokines. Compared with hUCMSC-CM-treated *NLRP1* knockdown cells, the hUCMSC-CM-treated *CASP4* knockdown cells showed reduced IL-1*α* secretion but increased IL-1*β* and IL-18 secretion (Figure 6(d)). These results are consistent with the results of western blotting, confirming that the noncanonical pathway was inhibited and hUCMSC-CM-induced pyroptosis mainly occurred via the caspase-1-mediated canonical pathway in *CASP4* knockdown MCF7 cells, whereas the canonical pathway was inhibited and hUCMSC-CM-induced pyroptosis mainly occurred via the caspase-4-mediated noncanonical pathway in *NLRP1* knockdown MCF7 cells.

## 4. Discussion

Breast cancer, the cancer that originates in the breast tissues, is divided into five types based on its molecular characteristics: Luminal A, Luminal B, HER2 enriched, basal like/triple-negative, and other special types of breast cancer [23]. The response to treatment and prognosis varies with the type of breast cancer. Therefore, cancer type-specific treatments are prescribed [24]. Our study focused on investigating the effect of hUCMSC-CM on breast cancer cell line MCF7. The MCF7 cells are Luminal A type, which is ER/PR positive and HER2 negative, with a high expression of ER-related genes and low expression of proliferation-related genes [25]. Typically, it is a low-grade cancer with an excellent prognosis.

MSCs show several promising applications in cell therapy and gene therapy owing to their unique characteristics. The relationship between MSCs and cancer has been studied widely by many research groups. Several studies have shown that the use of MSCs in cancer therapy is a two-edged sword that can suppress or promote cancer growth [26]. The different effects of MSCs on cancer growth depend on the source of MSCs and the type of cancer cells. Chen et al. [27] reported that aggressive ER-negative breast cancer cells show stronger ability to engulf MSCs than the aggressive ER-positive MCF7 cells and nontumorigenic MCF10A cells do and that such engulfment results in the development of breast cancer with enhanced migration, invasion, and metastatic properties.

MSCs interact with cancer cells via various mechanisms, including direct contact and subsequent engulfment of cancer cells [27, 28] and immunomodulation to influence the survival of tumor cells [29, 30]. Paracrine actions of MSCs might be crucial for their immunomodulatory functions. Extracellular vesicles (EVs) represent a group of cell-derived bilayered membrane structures that contain bioactive paracrine molecules, which can affect the target cells [31]. EVs are commonly classified into three subtypes: exosomes, microvesicles (MVs), and apoptotic bodies [32]. Recently, many studies reported that EVs derived from MSCs (MSC-EVs) can regulate cancer cell proliferation, angiogenesis, and metastasis. EVs derived from hUCMSCs were also reported to be effective against cancer cells. Wu et al. [33] found that hUCMSC-EVs might inhibit bladder tumor T24 growth by downregulating Akt protein kinase phosphorylation and upregulating cleaved caspase-3. Hendijani et al. [34] showed that the hUCMSC secretome displayed an antiproliferative effect on the leukemia cell line and exerted an additive cytotoxic effect in combination with doxorubicin. Usually, hUCMSC-CM can be used in preliminary studies investigating the effect of hUCMSC-EVs. He et al. [35] demonstrated that hUCMSC-CM inhibited cancer growth and radiosensitivity of the breast cancer cell line MDA-MB-231 by downregulating the Stat3 signaling pathway. Hong et al. [36] showed that hUCMSC-CM can decrease the cisplatin-induced apoptosis of oocytes and granulosa cells in a cisplatin-induced ovarian injury model. We previously reported that hUCMSC-CM could induce MCF7 pyroptosis in vitro, and our RNA sequencing studies revealed a significant increase in the expression of pyroptosis-related gene *NLRP1* and *CASP4* in pyroptotic MCF7 cells. Therefore, in this study, we further investigated the effects of these two genes on MCF7 cell pyroptosis induced by hUCMSC-CM.

We first assessed the gene and protein expression of caspase-1, caspase-4, and NLRP1 in MCF7 cells undergoing hUCMSC-CM-induced pyroptosis. Although the mRNA levels of these three genes increased, the protein levels showed a different trend. In particular, NLRP1 protein decreased significantly. During pyroptosis, NLRP1 interacts with the adaptor protein ASC to form an inflammasome complex. Similarly, we detected the expression and localization of NLRP1 and ASC in pyroptotic MCF7 cells via immunofluorescence and found that these two proteins colocalized in pyroptotic MCF7 cells, suggesting the formation and involvement of the inflammasome in hUCMSC-CM-induced pyroptosis in MCF7 cells.

Next, we investigated the role of caspase-4 and NLRP1 in hUCMSC-CM-induced pyroptosis in MCF7 cells. shRNA-mediated knockdown of *CASP4* or *NLRP1* in MCF7 cells resulted in a 50–70% reduction in the corresponding transcript levels (Supplementary2). Pyroptosis occurs via the canonical and noncanonical pathways. Caspase-1 is the key molecule involved in the canonical pathway, and NLRP1 recruits ASC and pro-caspase-1 to form the NLRP1 inflammasome or directly interacts with pro-caspase-1 to activate caspase-1 and induce pyroptosis [5]. Caspase-4 is the key molecule involved in the noncanonical pathway. In this study, deficiency of caspase-4 or NLRP1 could not completely block either of these two pathways. Therefore, studying the role of one pathway by blocking the other could not be performed in this study. Nevertheless, we found some interaction between these two pathways.

Cell morphology and cell death analysis using Annexin V-FITC/PI and LDH assays showed that levels of hUCMSC-CM-induced pyroptosis in *CASP4* knockdown and *NLRP1* knockdown MCF7 cells were not significantly different from that observed in the NC group. This indicates that inhibition of caspase-4 or NLRP1 could not inhibit MCF7 cell pyroptosis induced by the factors secreted by hUCMSCs. Therefore, to elucidate the mechanisms underlying these observations, we further investigated the changes in the expression of pyroptosis-related genes in the shRNA-transfected MCF7 cells.

Caspase-1, also known as IL-1*β*-converting enzyme, is responsible for maturation and secretion of the proinflammatory cytokines IL-1*β* and IL-18 [16, 37–39]. Caspase-1 dimerization and self-activation is induced by inflammasomes, which are composed of a PRR, the adaptor ASC, and pro-caspase-1 [38]. ASC interacts with PRRs and pro-caspase-1 through its pyrin domain (PYD) and recruitment domain (CARD), respectively. PRRs such as NLRP1, NLRP3, NLRC4, AIM2, and Pyrin, respond to microbial, environmental, and host-derived danger-associated molecular patterns; microbe-associated molecular patterns; and pathogen-associated molecular patterns [40]. Activated caspase-1 cleaves gasdermin D to promote membrane pore formation and pyroptosis [41, 42]. In humans, NLRP1 contains a PYD, a function to find domain (FIND), and CARD. Therefore, NLRP1 can directly activate procaspase-1 by interacting with it or indirectly by recruiting ASC and pro-caspase-1 to form the NLRP1 inflammasome, which activates caspase-1 [5]. However, agents that can selectively activate human NLRP1 inflammasomes have not yet been identified. We previously reported significantly high expression of *NLRP1*and *CASP1* during hUCMSC-CM-induced pyroptosis in MCF7 cells. These results suggest that some factors in the hUCMSC-CM interact with NLRP1 and activate caspase-1 to induce MCF7 cell pyroptosis. However, hUCMSCs secrete a vast array of molecules, and the precise factors that interact with NLRP1 remain unknown and further studies are warranted to identify these factors. In this study, we knocked down *NLRP1* to elucidate its role in hUCMSC-CM-induced pyroptosis. hUCMSC-CM treatment did not considerably change caspase-4 expression at both mRNA and protein levels and did not change the amount of cleaved CASP4 but increased pro-caspase-1e xpression in *NLRP1* knockdown MCF7 cells. *NLRP1* mutations have been reported to increase systemic amounts of caspase-1 in patients with arthritis and dyskeratosis [43]; our results are consistent with these findings. Furthermore, compared to pro-caspase-1 levels, the number of active ASC complexes was reported to be a more important limiting factor for caspase-1 maturation/release [21]. The secretion of IL-1*β* and IL-18 is positively correlated with the activity of caspase-1. Therefore, in *NLRP1* knockdown MCF7 cells, we found ASC complexes, cleaved-CASP1, and the levels of secreted IL-1*β* and IL-18 decreased. These results suggest that *NLRP1* knockdown partly inhibited the canonical pyroptosis pathway but did not affect the noncanonical pathway.

Caspase-4 detects cytoplasmic LPS and triggers the noncanonical pyroptosis pathway in humans. Caspase-5 has a synergistic effect with caspase-4 [36, 44]. The oligomerization and activation of caspase-4 and caspase-5 are triggered by binding of their CARDs with the lipid portion of LPS. However, the CARD of caspase-5 is 56% divergent from that of caspase-4, suggesting that caspase-5 binds and responds to lipids with specificities different from those of caspase-4 [45]. In fact, these two caspases play different roles in different cells. LPS stimulation induced processing of procaspase-5, but not of caspase-4, and mediated IL-1 release in monocytes [44]. Caspase-5 expression was undetectable in U937 cells, and ectopic expression of caspase-5 partially triggered inflammasome activation in response to *Escherichia coli* LPS but did not trigger inflammasome activation in response to *Francisella novicida* LPS [21]. Caspase-5 could not be detected in LPS-stimulated THP1 cells [6]. In contrast, caspase-4 played an important role in epithelial cell death during *Shigella* infection [46], and in pyroptosis and IL-1*α* secretion in human gingival fibroblasts in response to Td92, a surface protein of the periodontal pathogen *Treponema denticola* [20]. We previously demonstrated that hUCMSC-CM-treated pyroptotic MCF7 cells did not show significant changes in caspase-5 expression but showed significantly increased caspase-4 expression. Therefore, we hypothesized that one or more of the factors secreted by hUCMSCs interact with caspase-4 and trigger MCF7 cell pyroptosis. In this study, we found that hUCMSC-CM treatment did not considerably change pro-caspase-1 expression at both mRNA and protein levels but moderately increased NLRP1 expression and ASC speck formation and decreased cleaved-CASP4 level in *CASP4* knockdown MCF7 cells. Furthermore, the levels of secreted IL-1*α* were lower but the levels of IL-1*β* and IL-18 in the *CASP4* knockdown MCF7 cells were higher than those in the NC and *NLRP1* knockdown MCF7 cells. These results are consistent with the previous reports [21, 44] and suggest that the noncanonical pathway is partly inhibited and MCF7 cell death occurs mainly via the canonical pathway in the absence of caspase-4. Caspase-4-mediated cell death was reported to trigger NLRP3-dependent caspase-1 activation and secretion of IL-1*β* and IL-18 [6, 21, 47, 48]. In addition, canonical inflammasomes can control the activation of noncanonical inflammasomes [49], and blocking the noncanonical pathway alone may not be sufficient to change the susceptibility to infections [50]. Therefore, other mechanisms affecting the canonical pathway may be involved in mediating pyroptosis in *CASP4* knockdown MCF7 cells, and further research is essential to elucidate these mechanisms.

## 5. Conclusions

We previously demonstrated that the factors secreted by hUCMSCs could induce pyroptosis in the breast cancer cell line MCF7. Moreover, our previous RNA sequencing analysis showed that the expression of pyroptosis-related gene *NLRP1* and *CASP4* increased significantly. In this study, we elucidated the role of these two genes in hUCMSC-CM-induced pyroptosis in MCF7 cells. We found that although *CASP1*, *CASP4*, and *NLRP1* mRNA levels increased, the protein levels showed a different trend. In particular, NLRP1 protein decreased significantly. Further analysis for identifying the underlying reason revealed that NLRP1 interacts with ASC to form a complex, which is involved in MCF7 cell pyroptosis. Further investigation using *NLRP1* and *CASP4* knockdown MCF7 cells showed that knockdown of either *CASP4* or *NLRP1* could not rescue MCF7 cells from hUCMSC-CM-mediated pyroptosis. Further study on *CASP4*- or *NLRP1-*knockdown cells revealed that MCF7 cell pyroptosis occurred via both canonical and noncanonical pyroptosis pathways; when one pathway was inhibited, hUCMSC-CM induced MCF7 cell pyroptosis via the other pathway. Our study provides a foundation for further studies aimed at elucidating the precise mechanism underlying hUCMSC-induced pyroptosis in the breast cancer cell line MCF7 and aid the identification of potential therapeutic targets for breast cancer.

## Figures and Tables

**Table 1:** Sequences used for shRNA knockdown.

Name	Sequences
Control	5′- TTCTCCGAACGTGTCACGT-3′
CASP4-100	5′- GCCACTTAAGGTGTTGGAATC -3′
CASP4-265	5′- GCAACGTATGGCAGGACAAAT -3′
CASP4-1104	5′-GGAAGGTACAGCAATCATTTG -3′
CASP4-801	5′- GCCTCAGTCTGAAGGACAAAC -3′
NLRP1-2009	5′- GCAGGAAGGAATATTTCTACA-3′
NLRP1-1634	5′- GCTTCCAGCATGTCTTCTACT-3′
NLRP1-2523	5′- GCTAGAAGCATATGGAATACA-3′
NLRP1-630	5′- GCTTCTGCTCGCCAATAAAGC-3′

**Table 2:** Primer sequences for quantitative real-time polymerase chain reaction.

Gene name	NCBI ID	Primer sequences
CASP1	NM_033292	S: 5′TACAGAGCTGGAGGCATTTG 3′
		A: 5′GGACTTGCTCAGAGTGTTTCT 3′

NLRP1	NM_033004	S: 5′ATCTCATGCCTGCAACTACTC 3′
		A: 5′CTCTCGATACTGGTCCACAAAG 3′

CASP4	NM_001225	S: 5′GAATCTGACAGCCAGGGATATG 3′
		A: 5′CCATGAGACATGAGTACCAAGAA 3′

## Data Availability

The data used to support the findings of this study are included within the article and its supplementary information file.

## Supplementary Materials

Supplementary Materials Additional file 1: (a) the sequencing results of CASP4 shRNA vectors. (b) The sequencing results of NLRP1 shRNA vectors. Sequencing with T7 universal primers. Additional file 2: (a) gene expression of caspase-4. (b) Gene expression of NLRP1. MCF7 cells were transfected with shRNA vectors for 72 hours, and then, the total RNA was extracted from the cells. q-PCR was used to detect the target gene. NC: negative control. Data are presented as Ct (2^-*△△*Ct^) relative to negative control. Data are presented as mean ± S.D., *n* = 3.
