# Supplementary material for: The Role of Caspase-4 and NLRP1 in MCF7 Cell Pyroptosis Induced by hUCMSC-Secreted Factors
Source: Stem Cells Int. 2020 Jul 9;2020:8867115. doi: 10.1155/2020/8867115 (PMC7368222; doi:10.1155/2020/8867115)
Supplement: Supplementary Materials — Additional file 1: (a) the sequencing results of CASP4 shRNA vectors. (b) The sequencing results of NLRP1 shRNA vectors. Sequencing with T7 universal primers. Additional file 2: (a) gene expression of caspase-4. (b) Gene expression of NLRP1. MCF7 cells were transfected with shRNA vectors for 72 hours, and then, the total RNA was extracted from the cells. q-PCR was used to detect the target gene. NC: negative control. Data are presented as Ct (2-△△Ct) relative to negative control. Data are presented as mean ± S.D., n = 3. [file 8867115.f1.pdf]

a

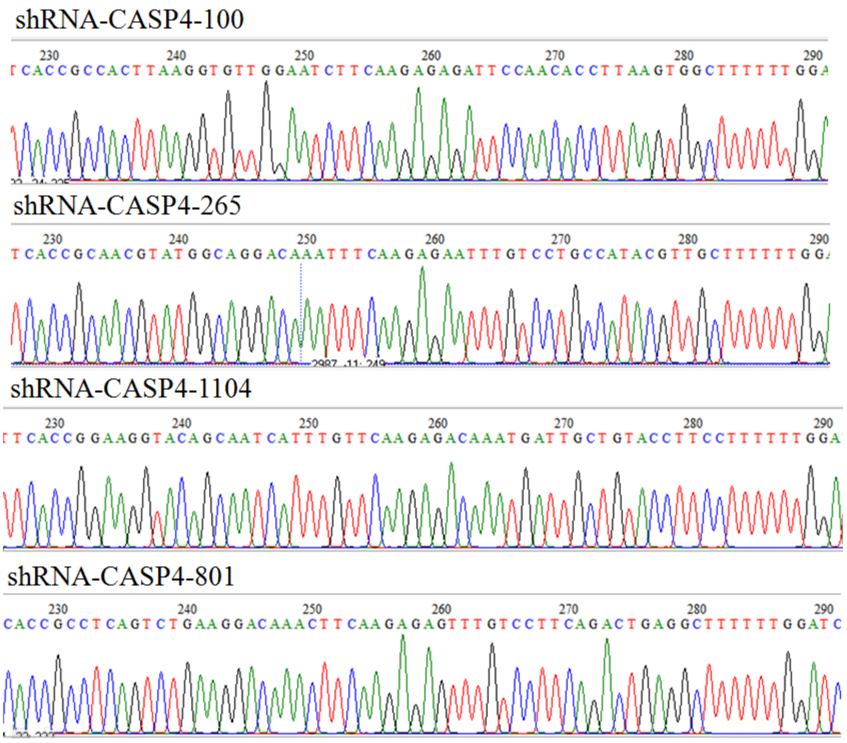

b

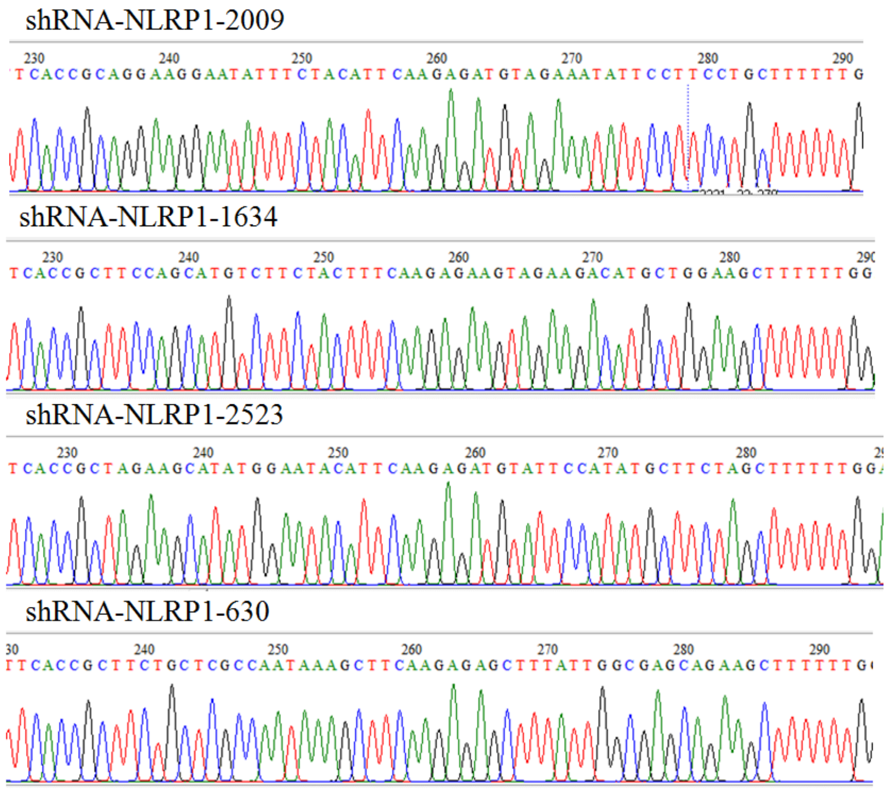

Additional file 1: (a) The sequencing results of CASP4 shRNA vectors (b) The sequencing results of NLRP1 shRNA vectors. Sequencing with T7 universal primers.

a

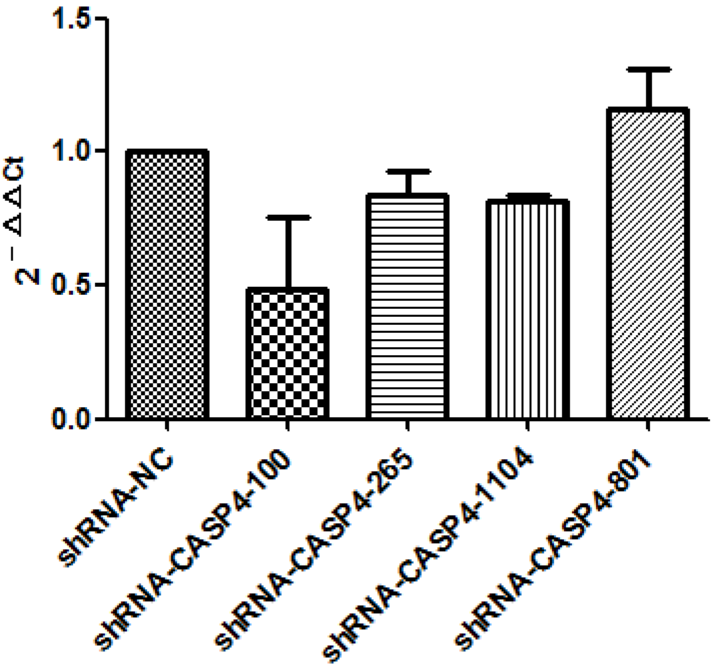

b

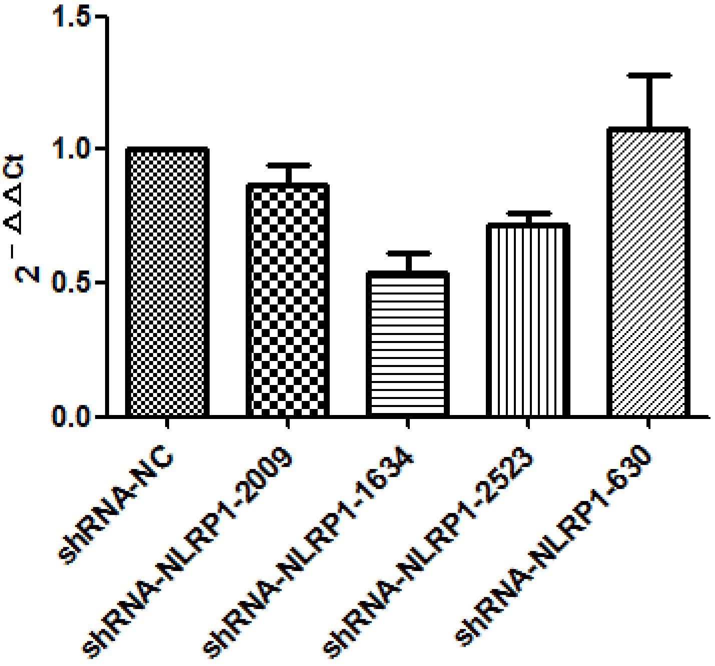

Additional file 2: (a) Gene expression of caspase-4. (b) Gene expression of NLRP1. MCF7 cells were transfected with shRNA vectors for 72 hours, and then the total RNA was extracted from the cells. q-PCR was used to detect the target gene. NC: negative control. Data are presented as $Ct^{(2-\triangle\triangle Ct)}$ relative to negative control. Data are presented as mean±S.D.. n=3.
